# Clinical and Biochemical Factors Associated with Infliximab Pharmacokinetics in Paediatric Patients with Inflammatory Bowel Disease

**DOI:** 10.3390/jcm14030845

**Published:** 2025-01-27

**Authors:** Ka Yu Wang, Omnia Salah Heikal, Patrick F. van Rheenen, Daan J. Touw, Arno R. Bourgonje, Paola Mian

**Affiliations:** 1 Department of Clinical Pharmacy and Pharmacology, University Medical Centre Groningen, 9713 GZ Groningen, The Netherlands; kayuwang@hotmail.com (K.Y.W.); heikalomnia@gmail.com (O.S.H.); d.j.touw@umcg.nl (D.J.T.); 2 Department of Paediatric Gastroenterology, Hepatology and Nutrition, Beatrix Children’s Hospital, University of Groningen, University Medical Centre Groningen, 9713 GZ Groningen, The Netherlands; p.f.van.rheenen@umcg.nl; 3 Department of Pharmaceutical Analysis, Groningen Research Institute of Pharmacy, University of Groningen, 9713 GZ Groningen, The Netherlands; 4 Department of Gastroenterology and Hepatology, University of Groningen, University Medical Center Groningen, 9713 GZ Groningen, The Netherlands; 5 The Henry D. Janowitz Division of Gastroenterology, Department of Medicine, Icahn School of Medicine at Mount Sinai, New York, NY 10029, USA

**Keywords:** inflammatory bowel disease, infliximab, pharmacokinetics, paediatric, covariates

## Abstract

Monitoring infliximab (IFX) concentrations is crucial for optimizing IFX therapy in children with inflammatory bowel diseases (IBDs) who show low response rates due to inadequate drug exposure. Substantial variation occurs in IFX trough concentrations in paediatric patients. **Objectives**: This study aimed to investigate IFX pharmacokinetics (PK) in children with IBD during both the induction phase and maintenance phases and to identify covariates associated with IFX PK. **Methods**: This single-centre retrospective cohort study was conducted at an academic children’s hospital. Data was extracted from paediatric IBD patients receiving IFX between January 2018 and October 2023 and included demographic-, clinical- and laboratory parameters collected from electronic health records. Linear mixed model analysis was performed to investigate associations between these parameters and IFX trough concentrations. Target attainment [≥15 μg/mL in induction or 5–10 μg/mL in maintenance phase] of the IFX dosing regimens was evaluated. **Results and Conclusions**: A total of 115 children (417 unique IFX concentrations) were included. Multivariate analysis revealed significant positive associations between IFX and albumin concentrations (β = 0.388, *p* = 0.010) and IFX concentrations with dose (β = 6.534, *p* < 0.001), and an inversion association between IFX concentrations and treatment phase (β = −4.922, *p* < 0.001). During the induction and maintenance phases, 57.2% and 30.6% of IFX concentrations were subtherapeutic, respectively. A systematic search of studies investigating factors influencing IFX concentrations was concurrently performed. Our findings were critically compared against existing literature to assess relevant clinical and biochemical determinants of IFX PK in children with IBD. Our findings highlight the need for personalized dosing strategies in pediatric IBD patients, particularly during the induction phase. By implementing therapeutic drug monitoring (TDM) and considering clinical and biochemical factors, clinicians can implement more personalized strategies, potentially improving treatment efficacy and reducing the risk of treatment failure or adverse effects. This approach could lead to better target attainment, potentially enhancing clinical outcomes and minimizing premature switching to other therapies.

## 1. Introduction

Inflammatory Bowel Diseases (IBDs) are chronic immune-mediated disorders that include Crohn’s disease (CD) and ulcerative colitis (UC). Paediatric onset of the disease occurs in about 10% of all cases. The incidence of IBD has increased rapidly over the past decades, especially in countries where the incidence used to be low [1]. Moreover, paediatric-onset IBD often presents with clinically greater severity compared to adult-onset cases, underscoring the need for targeted therapeutic strategies in this age group. Children with IBD are not just small adults with IBD as they differ significantly in phenotype, disease extent, and outcomes. Studies have shown that children often have more extensive IBD compared to adults [2,3]. Adults typically are characterized with left-sided UC whereas children and adolescents present more often with pancolitis [3,4,5]. In CD, adolescents have more extensive disease compared to adults as they present with ileocolonic disease and upper gastrointestinal involvement. Childhood-onset CD exhibited more extensive disease involvement compared to adult-onset CD (43.2% vs. 3.2%) [2]. Another study comparing disease progression in adults versus paediatric patients with IBD revealed that extensive colitis was observed in 82% of children with UC, nearly double the rate seen in adults (48%) [2]. Moreover, adolescents with IBD were more likely to have more severe disease which required early second-line immunomodulatory drugs or even escalation of therapy [3,6,7]. Given these variations in the severity and the extent of the disease in young patients, the urgency for developing tailor-made therapeutic strategies in this group is needed.

Treatment opportunities for patients with IBD have increased dramatically over the past 15 years, but the majority of biological agents and small molecules have not been authorized for use in children [8,9]. To date, only the anti-tumor necrosis factor (TNF) agents infliximab (IFX) and adalimumab are approved for use in children [10]. When treatment options were limited, the primary goal was to reduce clinical symptoms. Now, with the advent of anti-tumour necrosis factor (TNF) antagonists, such as IFX, therapeutic goals have expanded towards mucosal healing and endoscopic and histological remission, with the opportunity of modifying patients’ disease course. Currently, treatment of paediatric IBD is focused on key aspects including (1) elimination of symptoms and restoration of quality of life, (2) restoration of normal growth, and (3) prevention of disease complications [11].

IFX has been well-studied in clinical trials in children and has been approved by the regulatory authorities (Food and Drug Administration and European Medicines Agency) for the treatment of moderate-to-severely active CD and UC. In children with CD, clinical response rates to IFX are approximately 64% with 50% achieving remission after the fourth infusion, as reported in a previous study [12]. For children with UC, real-world data show that only 20–60% of patients achieve clinical remission with standard maintenance dosing of anti-TNF biologics [13]. Despite early clinical response rates to IFX, roughly 25–40% of initial responders will lose response over time (secondary non-response) while a significant subset of patients will require dose adjustment (intensification) to maintain clinical remission [14,15,16,17].

One of the major factors contributing to low response rates is inadequate drug exposure, often indicated by low IFX serum trough levels [18]. Insufficient exposure to IFX is the main cause of primary non-response and secondary loss of response in IBD [18]. Therapeutic drug monitoring (TDM) has been implemented in clinical practice to monitor serum trough levels and perform individual-specific dose adjustments when deemed necessary This may prevent premature switching to another therapy due to assumed loss of response [19]. However, substantial variation in IFX trough levels exists between and within patients, which primarily stems from significant clinical- and disease heterogeneity [19,20]. To address this, it is important to delineate potential physical, clinical, and laboratory parameters that may influence IFX PK.

Therefore, the aim of this retrospective study was to study IFX PK in children with IBD in both the induction and maintenance phases and to explore clinical, laboratory, and physical covariates influencing IFX PK.

## 2. Materials and Methods

### 2.1. Patient Selection

This was a single-centre pharmacokinetic study based on routinely collected electronic healthcare data at the University Medical Center Groningen (UMCG) in the Netherlands. We used the clinical data of children with IBD who were treated at the infusion centre between January 2018 and October 2023, and who were younger than 18 years of age at the time of the IFX measurement. We only included patient data when at least one IFX serum concentration was reported in the medical records within the period of interest. All children enrolled in this study had a confirmed endoscopic and histological diagnosis of CD or UC. The timing of the start of IFX therapy relative to the date of diagnosis was at the discretion of the treating physician. Patients were excluded from the cohort if they were 18 years or older at the time of first determination of IFX concentration, diagnosed with conditions other than CD or UC, lacking at least one measurement of IFX and an unknown date of first IFX administration. Due to the retrospective and descriptive nature of this study, the need to attain informed consent was waived by the UMCG medical ethics committee (METc 2023/182).

### 2.2. Clinical Characteristics

The following patient characteristics were extracted from the electronic health records: age, sex, weight, height, body mass index (BMI), diagnosis, disease duration, and smoking status. Furthermore, albumin, C-reactive protein (CRP), and antibodies-to-IFX (ATI) were included if collected within 7 days before or after the IFX measurement, based on data availability. For faecal calprotectin (fCal), a range of 3 months from the IFX serum concentration was considered. Medication use which could influence IFX PK was also extracted, including the concurrent use of corticosteroids (e.g., prednisolone) and immunomodulators (e.g., thiopurines (azathioprine, mercaptopurine) and/or methotrexate). The date of the first IFX administration, the date of the IFX measurement, the IFX serum concentration, and the treatment phase (either induction or maintenance) were also collected.

### 2.3. IFX and Antibody-Towards-IFX (ATI) Level Analysis

To assess IFX concentrations and ATI levels, blood samples were sent to Sanquin Diagnostics in Amsterdam, The Netherlands. This was to ensure the robustness of the assay by measuring it in an independent laboratory. Samples were stored at 2–8 °C if measured within 24 h of collection or frozen on dry ice if longer than 24 h. Quality control (QC) protocols were implemented to ensure measurement accuracy and reliability.

IFX was measured using an enzyme-linked immunosorbent assay (ELISA). The lower limit of quantification (LLOQ) and upper limit of quantification (ULOQ) of the assay is 0.03 μg/mL and 250 μg/mL, respectively [21]. ATI levels were assessed using an anantigen binding test, with results categorized into four groups; <12 AE/mL is not demonstrable, 12–30 AE/mL is demonstrable but not quantifiable, 30–875 AE/mL is both demonstrable and quantifiable and >875 AE/mL is demonstrable and higher than ULOQ [22]. In this study, patients were considered negative for ATI if levels were < 12 AE/mL and positive if ≥12 AE/mL. ATI levels are determined if the IFX level is lower than expected or when there is doubt about the effectiveness of the IFX treatment [23].

### 2.4. Statistical Analysis

Baseline demographic and clinical characteristics were presented as means ± standard deviation (SD), medians with interquartile ranges [IQR] or as numbers with corresponding percentages (%). Assessment of normality was performed visually using kernel density plots and normal probability (Q-Q) plots and statistically using the Shapiro-Wilk tests. Linear mixed model (LMM) analysis was performed to assess associations between physical-, clinical-, and laboratory parameters and serum IFX concentrations, enabling to account for repeated measurements of serum IFX concentrations. Variables of interest included age, sex, weight, height, BMI, IFX dose, IFX induction or maintenance phase, albumin, CRP and fCal concentrations. All these variables were first univariately analysed as fixed effects entered either as factors (to compare estimated marginal means [EMM] between subgroups) or as covariates (to analyse the linear trend) and together with a random effect over individual subjects. Covariate estimates were presented with beta-coefficients with standard error (SE) and 95% confidence intervals (CIs). EMMs were presented with their corresponding 95% CIs. In multivariate analysis, significantly associated variables (*p* < 0.1) from univariate analysis were selected and analysed to improve the overall model performance. Best-fitted models and covariance structures were determined based on log-likelihood statistics. Data analysis and visualization were performed using the SPSS Statistics software package (v.28.0) (SPSS Inc., Chicago, IL, USA). Two-tailed *p*-values ≤ 0.05 were considered statistically significant.

### 2.5. Literature Search

A literature search was conducted using PubMed in September 2024 using terms relating to “pharmacokinetics”, “infliximab” and “child”. The full search query can be found in Appendix A. Included studies were categorized into noncompartmental analysis (NCA) and population pharmacokinetic models (popPK) models.

## 3. Results

### 3.1. Clinical Characteristics of Participants

**Figure 1:**
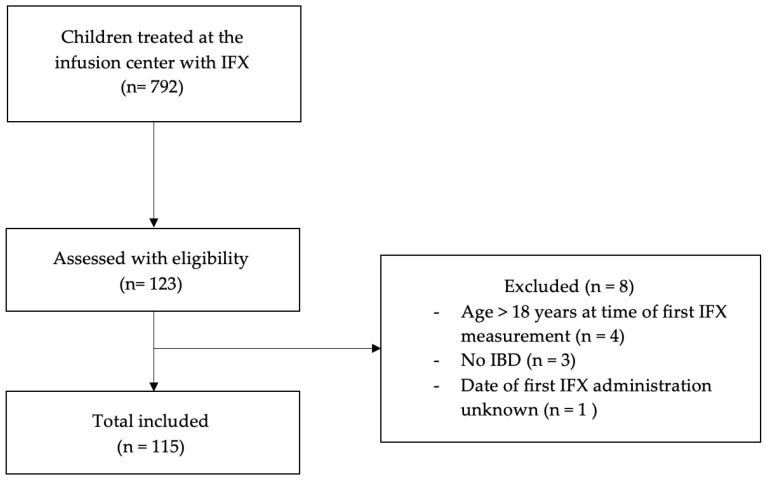
Flow diagram for patient selection in the retrospective study from January 2018 until October 2023. IFX: infliximab; IBD: inflammatory bowel disease.

Figure 1 shows that we included 115 patients who had at least one known IFX serum concentration. The baseline characteristics of this cohort are shown in Table 1. The study cohort consisted of 115 children, 51 (44.3%) of which were female and 64 (55.7%) male. The age of the population ranged between 5 and 17 years old (median 14.5 [IQR: 12–16] years). Patients had a wide range in body weights (19.2–107.8 kg; median 52.4 [IQR: 38.1–63.8] kg) and heights (110–194 cm; median 162.6 cm [IQR: 152.1–174.6]). In total, 77 patients (63.8%) received IFX 10 mg/kg, followed by 56 patients (33.3%) with 5 mg/kg, and 3 patients (2.4%) with 15 mg/kg. Only one patient (0.5%) received a dose of 8 mg/kg. A total of 417 IFX serum concentrations were obtained from the total patient population of which 260 and 157 samples were drawn during the maintenance and induction phase, respectively.

The distribution of IFX concentrations was significantly different across the two phases (*p* = 0.043). It was anticipated that patients would achieve remission and would be accompanied by lower IFX concentrations, as the value ranged from 0.03 to 45.0 μg/mL (median 8.05 [IQR: 4.4–14] μg/mL) and 0.005 to 74.0 μg/mL (median 11 [IQR: 3.05–25.0] μg/mL) for the induction and maintenance, respectively. Moreover, the CRP concentration significantly varied between the induction and maintenance phases as the median was 1.2 mg/L [IQR: 0.3–5.5] and 0.7 mg/L [IQR: 0.3–1.6], respectively (*p* < 0.001). The fCal concentration was significantly higher (*p* < 0.001) during induction compared to maintenance, indicating significant intestinal inflammation during induction and improvement of inflammation during maintenance. Out of 115 patients, 108 patients used concurrent medication. The majority (87.8%) used thiopurines (monotherapy azathioprine or mercaptopurine or a combination of azathioprine and mesalamine). Only 7 patients used methotrexate whereas the remaining patients did not use any co-medication. Besides IFX, 8 patients (7.0%) used prednisolone.

### 3.2. Target Attainment

The target concentration of IFX TDM for this patient population was based on the European Guidelines [24]. In total, 159 IFX serum concentrations were taken during the induction phase, of which 57.2% was subtherapeutic (<15 μg/mL) and 42.8% supratherapeutic (≥15 μg/mL). The target concentration is ≥15 μg/mL or 5–10 μg/mL in the induction or maintenance phase, respectively [24]. A total of 417 IFX serum concentrations were obtained from the total patient population. Based on the therapeutic window of 5–10 μg/mL in the maintenance phase, 40.7% could be considered as supratherapeutic (>10 μg/mL), 28.7% as therapeutic (5–10 μg/mL) and 30.6% as subtherapeutic (<5 μg/mL). In total, 159 IFX serum concentrations were taken during the induction phase, of which 57.2% were subtherapeutic and 42.8% therapeutic. A total of 258 IFX serum concentrations were obtained from the total patient population during the maintenance phase. Based on the therapeutic window of 5–10 μg/mL in the maintenance phase, 40.7% could be considered as supratherapeutic (>10 μg/mL), 28.7% as therapeutic (5–10 μg/mL) and the remaining 30.6% as subtherapeutic (<5 μg/mL).

### 3.3. Associations Between Clinical and Biochemical Factors and IFX Serum Concentrations

**Figure 2:**
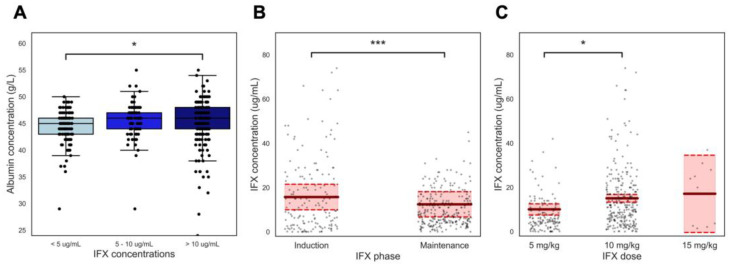
Relationships between albumin levels, treatment phase, dosage, and IFX concentrations (µg/mL). (**A**) shows albumin concentrations (g/L) compared between sub-, therapeutic-, and supratherapeutic ranges of IFX concentrations. (**B**) shows IFX concentrations compared between induction and maintenance phases, showing significantly lower levels during maintenance compared to induction. (**C**) shows IFX concentrations compared across different IFX doses (5 mg/kg, 10 mg/kg, 15 mg/kg), with an expected dose-dependent increase in average IFX concentrations. The boxes represent the interquartile range (IQR), and the whiskers extend to 1.5 times the IQR from the first (lower end) and third (higher end) quartiles. The bars and 95% confidence intervals are based on the estimated marginal means and its confidence intervals, derived from linear mixed models (LMMs). * indicates *p* < 0.05, *** indicates *p* < 0.001.

In univariate analysis, albumin (β = 0.388, *p* = 0.039), IFX dose (β = 6.534 and 9.424, *p* < 0.001, reference set at lowest dose of 5 mg/kg) and IFX treatment phase (β = −4.922, *p* < 0.001, reference set at induction) were observed to be significantly associated with IFX concentrations (Figure 2, Table 2). No apparent difference in IFX concentrations was observed across diagnosis subtypes (β = −0.088 *p* = 0.158, reference is CD), however, the majority of patients (93.9%) were diagnosed with either CD or UC. Hence, univariate analysis was performed excluding patients with IBD-Unspecified (IBD-U) and very early onset (VEO)-IBD while ASUC was classified under UC, but this did not materially change the result (*p* = 0.969). Lastly, none of the concurrent medications assessed—thiopurines, methotrexate or mesalazine—demonstrated a statistically significant effect on IFX concentration, *p* = 0.270, *p* = 0.555, *p* = 0.106, respectively.

In multivariate analysis, we combined the following variables, which appeared significantly associated with IFX concentrations from univariate analysis: albumin, IFX dose (excluding the dose of 8 mg/kg), IFX phase and diagnosis (only considering those diagnosed with CD or UC). This resulted in improved model performance (Δ-2LL = 33.790, *p* <0.001). In this multivariate model, the associations between IFX concentrations and albumin (*p* = 0.010), IFX dose (*p* < 0.001) and IFX treatment phase (*p* = <0.001) were all retained, whereas still no significant difference across diagnosis subtypes was identified (*p* = 0.928).

## 4. Discussion

The aim of this study was to identify physical-, clinical-, and laboratory parameters associated with serum IFX concentrations in children with IBD. We found significant associations between IFX serum concentrations and albumin concentrations (*p* = 0.010, β = 0.388), IFX dose (*p* = <0.001, β = 6.534 for 10 mg/kg and 9.424 for 15 mg/kg; reference set at the lowest dose of 5 mg/kg) and IFX induction or maintenance phase (*p* < 0.001, β = −4.922; reference set at induction).

We performed a systematic search of existing literature to compare our results with existing literature (Table 3). With the search, we aimed to find studies describing covariates influencing the PK of IFX in children. In total, 30 studies were included and separated into noncompartmental analysis (NCA) (n = 17) and population PK models (n = 13). The magnitudes of the included covariates explaining variability within the PK parameters are also described in Table 3.

### 4.1. Albumin

Albumin exhibited a positive correlation with IFX concentrations in our study, suggesting that higher albumin levels may increase IFX concentrations. This aligns with the findings from our systematic literature search (Table 3). Here 13 studies (3 of which were NCA and 10 were popPK models) identified a positive correlation between albumin and IFX concentrations [18,25,26,27,28,29,30,31,32,33,34,35,36]. In NCA studies, Clarkston et al. [27] conducted univariate regression analysis for treatment outcomes and found that serum albumin concentrations ≤3.5 g/dL before the start of IFX treatment were associated with IFX concentrations <18 μg/mL at the third infusion. Naviglio et al. [26] demonstrated that IFX concentrations of 3.11 μg/mL at the fourth infusion (cut-off value for sustained remission) were correlated with lower albumin levels (*p* = 0.03) and higher CRP (*p* = 3.6 × 10^−5^) and fCal levels (*p* = 0.0014) in logistic regression analysis. Additionally, IFX concentrations were directly correlated with albumin levels in the linear mixed effects model conducted by the study (*p* = 0.0033). Similar findings were also reported by Rolandsdotter et al. [25] in their linear regression model (*p* = 0.0005, r^2^ = 0.2182).

Previous popPK studies have shown that low concentrations of albumin are linked to increased clearance (CL) of IFX. [18,28,29,33,34,35,36,37,38]. The weight of albumin as a covariate on CL was reported in 10 out of 13 popPK models and ranged from −2.33 to −0.445. The consistent identification of serum albumin as a significant covariate in IFX PK across multiple studies underscores its importance in clinical practice. By incorporating albumin levels into dosing considerations, clinicians may achieve better dose optimization.

The statistical association between albumin concentrations and IFX concentrations may be explained by the involvement of FcRn [37]. The FcRn plays a crucial role in protecting both albumin and IgG antibodies (including IFX), resulting in extending the half-life of both by preventing their degradation in lysosomes [39]. Higher albumin concentration may reflect an increased number of FcRn receptors, contributing to the preservation of albumin in the bloodstream. Additionally, since FcRn protects IFX from catabolism, an increased number of FcRn receptors would likely result in a reduced elimination of IFX [37]. This mechanism provides a plausible explanation for the observed association between elevated albumin levels and slower clearance of IFX.

Moreover, albumin and monoclonal antibodies also share molecular similarities, such as comparable molecular weights (±66.5 kDa and ±150 kDa for albumin and IgG antibodies respectively) and their primary distribution within the plasma [40,41,42]. In inflammatory diseases such as IBD, albumin may serve as a surrogate for mAbs like IFX due to their overlapping behaviours, especially in uncontrolled inflammation. Inflammatory states are typically associated with hypoalbuminemia, therefore this phenomenon likely contributes to the observed need for higher IFX dosing in patients with elevated inflammatory burden. Thus, while albumin itself does not play a direct mechanistic physiological role in modulating IFX PK, its concentration may serve as an indicator of the inflammatory state and FcRn-mediated preservation of IFX.

Baseline albumin levels are easily obtainable through routine blood samples and therefore may serve as a predictor of IFX disposition in patients. This approach is particularly useful as it is available prior to IFX administration, enabling clinicians to predict drug dispositions before treatment. In contrast, the presence of ATI can only be detected after drug administration. Therefore, the monitoring of baseline albumin concentration offers a proactive method for optimizing IFX therapy. For patients with low baseline albumin levels, clinicians may anticipate faster IFX clearance and consider adjusting the dosing schedule or consider additional strategies such as closer therapeutic monitoring to ensure optimal treatment [37]. Patients with low albumin levels may require higher initial or more frequent dosing of IFX to achieve and more importantly, to maintain therapeutic levels [43].

Furthermore, albumin monitoring facilitates personalized treatment approaches. Clinicians can predict which patients are more likely to need dose escalation. This allows adjustments in treatment plans, thereby potentially improving outcomes and reducing the risk of treatment failure [44].

The incorporation of serum albumin as a covariate in the development of model-informed precision dosing tools for IFX can guide personalized dosing strategies. The association between IFX and albumin further underlines albumin’s relevance as a biochemical factor.

It should be noted that 4 NCA studies found albumin to be a nonsignificant predictor of IFX concentration at the 6th, 14th, and 22nd weeks of IFX treatment [45,46,47]. In the analysis by Levy et al., [48] the study found that demographic variables including age, weight, and albumin levels were not significantly associated with changes in IFX concentrations. The popPK model by Maximonova et al. did not implement albumin as a covariate in their model as they did not find it significantly improved model performance [49].

### 4.2. Treatment Phase

The association between the treatment phase and IFX serum concentrations was also highlighted in our study, showing distinct patterns between the induction and maintenance phases. Overall, IFX concentrations were significantly higher during the induction phase compared to the maintenance phase in children. This finding is consistent with our expectations, as the dosing regimen during the induction phase typically involves higher IFX doses and shorter intervals (weeks 0, 2, and 6), resulting in higher serum IFX concentrations [50].

The same trend is observed in the literature; Adedokun et al. reported median IFX concentration at weeks 2 and 6 of 19.3 and 14.5 μg/mL, respectively, compared to 1.9 μg/mL at week 30 [51]. The study by Hämäläinen et al. also reported higher median IFX concentrations at induction (17.6 mg/mL, range 0–48 µg/mL) compared to the maintenance phase (3.55 µg/mL). Previous studies mentioned the difference in concentrations during induction versus maintenance phases also highlights the importance of TDM as IFX concentrations below 29 μg/mL at the second infusion (OR = 13.1; *p* = 0.016) and 18 μg/mL at the third infusion (OR = 11; *p* = 0.03) are associated with a higher chance of clinical and biological nonresponse [27,52]. Vermiere et al. found that IFX concentrations above 10 μg/mL at the fourth infusion were significantly associated with shorter time to remission and better clinical outcomes (HR = 1.5; *p* < 0.01) [52]. Throughout the maintenance phase, IFX concentration ≥ 3.5 μg/mL was associated with sustained remission (adjusted OR = 0.13; *p* = 0.01) [26,45]. Maintaining adequate IFX concentrations is crucial for sustaining long-term remission. Contrary to the previous studies, Dipsaquale et al. [53] noted that IFX concentration was not significantly associated with combined clinical and biochemical remission at fourth INF (OR = 0.01; *p* = 0.819) or sixth INF (OR = 0.017; *p* = 0.0732). PopPK models did not include the treatment phase as a covariate.

### 4.3. IFX Dose

IFX dose was positively and significantly associated with IFX serum concentrations in our study. Two studies (Table 3) found a positive association between IFX dose and concentration [28,45]. For example, Adedokun et al. demonstrated that doubling the starting dose of 5 mg/kg to 10 mg/kg in children with UC increased the median concentration from 1.9 μg/mL to 2.9 μg/mL, also supporting the positive correlation between IFX dose and IFX concentration [51]. Hämäläinen et al. found that at week 2 (*p* < 0.001) and week 6 (*p* = 0.0445) of treatment, IFX concentrations were positively associated with the total amount of IFX given at the first infusion (mg). Indicating that children with lower body weight would have lower IFX concentrations [54]. Chi et al. conducted a study to determine whether combination therapy significantly affected IFX concentration. They reported that patients taking higher doses of IFX (≥10 mg/kg) had significantly higher IFX concentrations than patients taking lower doses of IFX (<10 mg/kg) and within each of those groups patients on combination therapy had higher IFX concentrations than patients who are not [45].

This study did not test the correlation between dose interval and IFX concentration. However, two NCA studies found dose interval to be negatively correlated with IFX concentration, shortening the interval resulted in increased concentration [48,54]. The study by Levy et al. reported that for every 10% decrease in interval, IFX concentration was increased by 1.6 μg/mL (*B* = 1.6, *p* < 0.001).

Interestingly, Levy et al. found that dose increase did not cause a predictable increase in IFX concentration (*p* = 0.9) [48]. Other studies also found no significant correlation between dose and IFX concentrations [25,47,54]. Jongsma et al. [47] only reported a non-significant difference in IFX concentrations between different IFX dosages (*B* = 0.050, *p* = 0.051). The study by Rolandsdotter et al. found that changing the dose (mg/kg/day) did not cause a change in IFX concentration (*p* = 0.58) The present data, however, reinforce the notion that therapeutic IFX concentrations can be achieved by increasing the dose per kg or by implementing decrease dosing intervals [28]. In general, younger children may require higher doses compared to adolescents due to their increased CL [55].

### 4.4. Other Covariates

In our study, we did not identify age in association with IFX concentrations. Three previous studies with varying age distributions are in concordance with this observation (Table 3) [32,34,47]. However, it should be acknowledged that the age distribution within our cohort deviates from a normal distribution, thereby impacting data interpretation. The majority of our patient population is older than 12 years, therefore resulting in a relatively small sample size of children younger than 13 years. Consequently, definitive conclusions on the absence of an association between age and IFX concentration cannot be drawn from our study. In contrast to our findings, Dipsaquale et al. found age to be positively associated with IFX concentration (*B* = 1.950, *p* = 0.048), but their study included a younger age range compared to our study [53].

Similarly, body weight (which was normally distributed in our study) was also not significantly associated with serum IFX concentrations in our study. Several other studies have looked into the correlation between IFX CL and body weight (Table 3). One study, by Jongsma et al. also reported no clear relationship between body weight and IFX concentration in young children with IBD [47]. The absence of an association between body weight and IFX exposure in children may be related to the narrow weight range (median 52.4 [IQR: 38.1–63.8] kg).

Contrary to our findings, body weight is commonly used as a covariate in popPK models on IFX. 9 literature popPK models report body weight as a significant covariate affecting IFX TL [28,29,30,31,32,33,36,49]. The study by Adedokun et al. [51] mentioned that the dose needs to be adjusted by body weight to account for the size difference between adults and children. Systemic exposure to infliximab was predicted to be approximately 40% lower in children between 2 and 6 years [51]. Lower drug exposure in younger children is due to differences in body weight, rather than age itself as it was not a significant covariate once body weight was accounted for. The relationship between CL and peripheral volume of distribution (Vd) with body weight is described as non-linear. Therefore, a lower body weight tends to have increased CL and Vd per kg. Using a standard linear dosing regimen of 5, 10 or 15 mg/kg and the non-linear relationship between body weight and IFX CL may predispose younger children to inadequate exposure to IFX [51].

Our findings indicated no significant association between IFX concentration and the use of any concurrent medication. In contrast, a cross-sectional study of paediatric patients observed that concomitant use of MTX increased IFX trough levels compared to those who only received IFX monotherapy. (15.59 μg/mL and 12.35 μg/mL, respectively) [45]. Similarly, a study by Vermeire et al. showed that adults not taking concurrent immunosuppressive therapy such as AZA or MTX had lower IFX levels (median 2.42 μg/mL) compared to those taking immunosuppressive therapy (median 6.45 μg/mL), although this difference did not reach statistical significance (*p* = 0.065) [56]. Despite the lack of statistical significance in our study, existing literature shows the importance of combining an immunosuppressant with IFX [57,58,59]. Both AZA and MTX have been shown to maintain IFX levels and reduce the formation of ATI [47,56]. The incidence of antibodies to IFX in our study however was low and may explain the absence of impact of concurrent medication on IFX PK. In terms of which add-on medications should be used, in most situations there is no preferred immunosuppressive. Previous studies showed that both AZA and MTX could be effective in enhancing IFX PK [56,57].

### 4.5. CRP

Finally, the present study also did not demonstrate a significant correlation between serum IFX concentrations and CRP levels. This is in contrast to existing literature (Table 3) [25,26,46,60,61,62]. Notably, the observation of diminished serum IFX concentrations is seen in patients with high CRP, suggesting a higher rate of elimination of the drug is associated with systemic inflammation [29,33]. This was confirmed by several studies (5 NCA, 2 popPK models) in children in which serum IFX concentrations showed a significant negative correlation with CRP concentrations [25,26,27,29,35,45,61]. This may be due to the lower CRP levels observed in the patients of this study compared to those in previous studies.

Our study examined a comprehensive range of potential factors influencing IFX PK. Among these, albumin, treatment phase and dose were significantly associated with IFX concentrations. These findings highlight the robustness of our approach, which systematically evaluated multiple factors that could influence dosing strategies. Although the other covariates did not demonstrate significant associations with IFX PK, their lack of significance remains clinically meaningful. This suggests that these variables might not need to be accounted for by dosing adjustments or model-based approaches. The breadth of testing different covariates enhances the study’s impact by clarifying which variables are unlikely to require consideration in future clinical applications.

### 4.6. Target Attainment

In addition to identifying factors associated with serum IFX concentrations, it is equally important to assess whether the current dosing regimen achieves the desired therapeutic targets. Achieving an optimal therapeutic range of IFX concentrations has been associated with improved clinical outcomes in paediatric patients with IBD [25,63]. This can be accomplished by tailoring dosing regimens to individuals using therapeutic drug monitoring (TDM)-guided management and model-informed precision dosing (MIPD). MIPD employs mathematical and statistical methods to customize dosage regimens, potentially leading to appropriate therapy adjustments and increased remission rates [64,65]. Clinical trials conducted in adults have established the efficacy of IFX at a target concentration exceeding 3 μg/mL [66,67,68]. While the optimal target concentration for paediatric patients remains undefined, the therapeutic drug monitoring (TDM) module of the Dutch Inflammatory Bowel Disease guideline for children introduces a concept that stratifies the target concentration based on the phase of IFX therapy. The target for the induction phase is generally accepted as ≥15 μg/mL to achieve adequate mucosal healing. In our study, 57.2% of the serum IFX concentrations measured during induction therapy were subtherapeutic. This low rate of target attainment compromises the ability to achieve remission early in the treatment.

The therapeutic range set during the maintenance phase is 5–10 μg/mL. Consequently, approximately 30% of IFX concentrations were classified as subtherapeutic. This observation raises the possibility that the standard dose of 5 mg/kg administered every 8 weeks may be inadequate for this subset of patients to attain therapeutic concentrations. Thus, consideration may be given to adjusting either the dose or the dosing interval to ensure therapeutic IFX concentrations within the therapeutic window [28]. These findings align with previous paediatric studies which report that standard weight-based dosing often results in suboptimal therapeutic IFX concentrations during maintenance [63,69]. Van Hoeve et al. found that most pediatric patients have subtherapeutic IFX concentrations postinduction on the current standard IFX regimen [69].

Bauman et al. [28] found that a starting dose of 5 mg/kg (administered every 8 weeks) resulted in subtherapeutic IFX concentrations in the majority of patients, with only 24.2% achieving a target trough level of ≥5 μg/mL. When shortening the interval to every 4 weeks in the simulated patient population, the target attainment increased to 84.4% whereas doubling the dose to 10 mg/kg only increased the target attainment to 56.2%. This suggests that IFX concentration and drug exposure are more influenced by the dosing interval than by the dose itself. Similar trends were observed in two retrospective paediatric cohort studies in which shortening of the dosing interval resulted in more patients reaching the IFX target concentration of >3 μg/mL compared to increasing the dose [70,71]. Seven studies [25,26,27,28,34,61,62], as shown in Table 3, have looked into the development of infliximab target concentrations or clinical/biological remission in paediatrics. Clarkston et al. found that an IFX concentration ≥18 μg/mL at week 6 and >5 μg/mL at the start of maintenance were associated strongly with clinical and biological response [27]. Whereas Ungar et al. reported IFX targets of 9.2 μg/mL at week 2 and 7.2 μg/mL at week 6 during induction in order to achieve clinical remission [61]. Moore et al. reported children in the higher TL quartiles had increased rates of clinical, biological and combined remission (*p* = 0.04, *p* < 0.001 and *p* = 0.01, respectively) [62]. The use of immunomodulators as combination therapy with IFX therapy has been associated with higher median IFX trough levels and is shown to reduce the formation of ATIs compared to IFX monotherapy. However, in clinical practice, their use is weighed against the risk of increased adverse events associated with the use of immunomodulators [24,72].

Our data reinforce the importance of TDM to identify suboptimal IFX concentrations and achieve dose optimization. Dose intensification through increasing the dose or shortening the dosing intervals may be useful to improve target attainment and achieve clinical remission.

### 4.7. Limitations

Our study has several limitations that should be acknowledged. Firstly, the effect of ATI on IFX concentrations could not be analysed in this cohort due to only being found in one patient. ATIs have been implicated as a potential cause of poor IFX therapy response, leading to treatment discontinuation and switching to alternative treatments. Moreover, it is important to acknowledge that the current study included a relatively small cohort and has a retrospective design. The latter introduces unavoidable biases such as recall bias and missing data. Not all requisite data for the study could be extracted from the database. For instance, pharmacodynamic data such as therapeutic outcomes and information on patient condition were not systemically registered and could therefore not be reliably extracted from the electronic health records due to the retrospective nature of this study. Instead, a prospective study needs to be established to better characterize exposure-response relationships for this group of patients. Physical examination was also not performed routinely, hence lacking measurements such as body weight and height. Additionally, the absence of routine endoscopic evaluations during the follow-up of paediatrics limited our ability to accurately assess disease severity. Endoscopy is considered the gold standard for evaluating the activity but was only performed shortly before starting IFX therapy. Consequently, the impact of endoscopic findings on the treatment remains unclear. A strength of our study is multiple IFX concentrations per patient were collected, if available in the patient’s dossier. This resulted in 417 IFX concentration samples.

### 4.8. Future Research

Despite the insights gained from the current study, several avenues for future research warrant further exploration. Prospective studies with larger cohorts and longitudinal follow-up are needed to confirm and elucidate our findings. Future prospective studies could also benefit from linking the pharmacokinetic data to therapeutic outcomes to increase the clinical utility of the findings. Moreover, incorporating routine endoscopic evaluations throughout treatment could provide valuable insights into the relationship between IFX therapy and disease development [73,74]. Finally, exploring alternative dosing strategies such as personalized dosing based on albumin concentrations may provide optimization of IFX therapy in paediatric patients with IBD.

**Table 3:** Overview of non-compartmental and population-PK model analysis on investigating covariates influencing the PK of IFX in children.

Author (year) [citation]	Drug	Administration	Study Design	N	Age and Weight	Disease State	Covariates	Magnitude of Influence of Covariates	Conclusion
Non-compartmental analysis									
Vermiere et al. (2024) [52]	IFX	1. STD 2. STD induction + start maintenance with PK and clinical outcome of induction 3. PRO	Three retrospective cohorts	307	^a^ 16.7 (13–27) yrs ^a^ 54 (41–66) kg at INF 3	CD, UC	IFX TL, ATI, ALB, WT	Logistic regression and Kaplan Meier analysis with hazard ratio calculation IFX TL above 15 μg/mL at third INF increased likeliness of having CRP-based clinical remission in maintenance phase. (OR = 2.5, 95% CI 1.2–4.2) (*p* < 0.01) IFX conc. above 10 μg/mL at fourth INF were significantly associated with higher rates of remission (HR = 1.5, 95% CI: 1.9–3.6) and shorter time to remission (clinical and biochemical) than IFX conc. Below 10 μg/mL (*p* < 0.01) Forecasted IFX conc. above 10 μg/mL at fourth INF in patients also resulted in 3.9-fold increased chance of sustained remission in maintenance phase. (OR = 3.9 95%, CI 2.3–6.5) (*p* < 0.01)	Analysing performances of PK dosing tool. Data indicates that forecasted IFX levels indicate suboptimal IFX levels could affect clinical outcome
Dipsaquale et al. (2023) [53]	IFX	STD	Prospective cohort	55	^c^ 10.6 ± 3.5 yrs WT: NR	CD, UC		Univariate and multivariate regression models Age at diagnosis was positively correlated to IFX TL (*B* = 1.950, 95% CI 0.019, 3.882) (*p* = 0.048) PCDAI/PUCAI negatively correlated to IFX TL at 4th INF (*B* = −0.401, CI −0.738, −0.064) (*p* = 0.023) Haemoglobin levels were positively correlated with IFX TLs at sixth INF (*B* = 1.853, 95% CI: 0.501–3.204) (*p* = 0.011) Presence of ATI correlated with lower IFX TL (*p* = 0.003) IFX TLs were not significantly associated with combined clinical and biochemical remission at fourth INF (OR = 0.01, 95%: 0.928–11.099) (*p* = 0.819) or sixth INF (OR = 0.017, 95%: 0.924–1.119) (*p* = 0.0732)	IFX dose/kg is positively correlated to IFX TL (*p* = 0.032) Concomitant treatment with other maintenance drugs resulted in higher TL in patients (*p* = 0.001)
Levy et al. (2023) [48]	IFX	STD	Retrospective cohort	86	IFX ^a^ 13.6 (11.8–15.7) yrs WT: NR	CD, UC	Dose and interval changes on TL	Generalized estimating equation Interval increase was negatively associated with IFX TL. For every 10% increase, TL decreased by 0.66 μg/mL (*B* = 0.66, 95% CI: 0.2–1.2) (*p* = 0.01) and for every 10% decrease in interval, TL increased by 1.6 μg/mL (*B* = 1.6, 95% CI: 0.9–2.4) (*p* = 0.01) Presence of perianal disease was negatively associated with increase in IFX TL and resulted in a median reduction of 2.5 μg/mL in TL after interval decrease. (OR = 2.4, 95% CI: 1.1–3.9) (*p* < 0.001)	Trough concentration response to IFX dose and interval changes are variable
Dubinsky et al. (2023) [75]	IFX	1. STD 2. PRO	2 prospective cohort studies	145	Standard ^a^ 13 (10–14) yrs ^a^ 39.1 (29.5–52.9) kg Proactive ^a^ 13.5 (11–15) yrs ^a^ 41.5 (30.3–53.0) kg	CD	Treatment group (standard/proactive), predictive factor of pharmacokinetic origin (IFX conc and baseline clearance)		Lower baseline clearance and proactive dosing are associated with increased disease control during induction Higher IFX conc. and lower clearance during induction and first maintenance INF increased disease control in maintenance phase IFX conc. And clearance together are better predictors of therapeutic outcome than either alone
Dubinsky et al. (2022) [76]	IFX	INF 1 and 2 STD PRO from INF 3	Prospective single arm intervention trial	156	5 mg/kg ^a^ 14.1 (11.3–16.7) yrs ^a^ 44.5 (30.5–66.1) kg 10 mg/kg ^a^ 15.3 (12.6–18.1) yrs ^a^ 49.8 (39.5–63.1) kg	CD, UC	CRP, WT, ALB, IFX dose, IFX TL, ATI		
Salvador-Martin et al. (2021) [77]	IFX	1. STD 2. Intensified dosing: - >10 mg/kg q8w - 5 mg/kg q4w	Observational, cross-sectional cohort	154	^a^ 12 (3–17.5) yrs WT: NR	CD, UC	DNA variants rs5030728 (TLR4) and rs11465996 (LY96)	Linear-by-linear association Fisher exact (univariate associations) rs5030728 (TLR4) and subtherapeutic IFX levels (OR = 3.434, 95% CI = 1.354–8.714) (*p* = 0.020) rs11465996 (LY96) and subtherapeutic IFX levels (OR= 0.241, 95% CI: 0.098–0.592) (*p* = 0.006)	DNA variant rs5030728 (TLR4) was positively associated with subtherapeutic IFX levels DNA variant rs11465996 (LY96) was negatively associated with subtherapeutic IFX levels
Jongsma et al. (2020) [47]	IFX	STD	Retrospective case control study	215	<10 yrs: ^a^ 8.32 (6.95–8.93) yrs >10 yrs ^a^ 14.32 (12.79–15.6) yrs WT: NR	CD, UC, IBD-U	IFX dose and interval, ATI	Linear mixed model More intensive treatment regimen, accounted to shorter intervals and IFX TL (β = − 0.006, 95% CI: 0.010 − 0.001) (*p* = 0.011) ATI positivity and IFX TL (β = − 0.681; 95% CI: 0.446–0.914) (*p* < 0.001)	More intensive treatment regimen, accounted to shorter intervals was positively associated with IFX TL. ATI positivity was negatively associated with IFX TL.
Moore et al. (2020) [62]	IFX	STD	Retrospective study	90	Severe UC ^b^ 13.6 (6.5–18.2) yrs Moderate UC ^b^ 14.9 (6.4–21.2) yrs WT: NR	UC	PUCAI ≥ 65 at start of IFX treatment and Time interval from the last IFX (days)	Spearman correlation coefficient PUCAI ≥ 65 at start of IFX treatment and IFX TL (β = −6.57, 95% CI −11.87 to −1.27) (*p* = 0.016) Time interval from the last IFX and IFX TL (β=−0.25, 95% CI: −0.45 to −0.05) (*p* = 0.0167)	PUCAI 65 at start of IFX treatment is negatively associated with IFX TL. Time interval from the last IFX is negatively associated with IFX TL.
Choi et al. (2019) [46]	IFX	STD	Retrospective analysis	103	^b^ 14 (13.3–17.5) yrs WT: NR	CD	ESR (cutoff value of 18 mm/hr)	Spearman correlation coefficient ESR and IFX TL during maintenance (spearman correlation coefficient, −0.11; *p* = 0.0005).	ESR is negatively associated with IFX TL.
Clarkston et al. (2019) [27]	IFX	NR	Sub-analysis of the PROSE study	72	^c^ 13.6 (4) yrs WT: NR	CD	prednisone, BMI, ESR, CRP, ALB, INF 2 IFX <29 ug/mL	Univariate regression analysis IFX conc. <29 μg/mL at INF 2 is associated with 13 times higher chance of clinical nonresponse (OR = 13.1, 95% CI: 2.4–246) (*p* = 0.016) Patients IFX conc. <18 μg/mL at INF 3 have a 6 times higher chance of developing clinical nonresponse (OR = 6.2, 95% CI: 1.5–4.2) (*p* = 0.024) Pre-IFX prednisone and biological non-response (OR = 3.9, 95% CI: 1.1–15.5) (*p* = 0.04) IFX conc <18 μg/mL at INF 3 is associated with biological nonresponse (OR = 11, 95% CI: 1.8–218) (*p* = 0.03) Pre-IFX prednisone is associated with IFX conc <29 μg/mL at INF 2 (OR = 4, 95% CI:1.3–13.1) (*p* = 0.018) BMI < 18 kg/m^2^ is associated with IFX conc. <29 μg/mL at INF 2 (OR = 4.9, 95% CI: 1.6–17.5) (*p* = 0.01) Use of pre-IFX prednisone is associated with IFX conc <18 μg/mL at INF 3 (OR = 4.8, 95% CI: 1.6–16) (*p* = 0.008) BMI < 18 kg/m^2^ before first IFX dose is associated with IFX conc < 18 μg/mL at INF 3 (OR = 3.6, 95%CI: 1.2–11.9) (*p* = 0.029) Pre-IFX ESR ≥ 20 mm/hr is associated with IFX <18 μg/mL at INF 3 (OR = 3.9, 95% CI: 1.1–15.9) (*p* = 0.04) CRP ≥ 0.5 mg/dL before first IFX dose is associated with IFX conc. <18 μg/mL at INF 3 (OR = 3.9, 95%CI: 1.1–15.4) (*p* = 0.04) ALB ≤ 3.5 g/dL before first IFX dose is associated with IFX conc. <18 μg/mL at INF 3 (OR = 5.4, 95%CI: 1.6–19.1) (*p* = 0.007) IFX conc. <29 μg/mL at INF 2 is associated with IFX conc. <18 μg/mL at INF 3 (OR = 17.8, 95%CI: 4.7–8) (*p* < 0.001)	
Naviglio et al. (2019) [26]	IFX	STD	Observational Cohort Study	49	^a^ 14.4 (11.6–16.2) yrs WT: NR	CD, UC	CRP, fCal	Multivariate logistic regression model and linear mixed effect model Patients who had IFX levels of 3.11 μg/mL at week 14 had a higher chance of achieving sustained remission at 54 weeks compared to patients with lower TL (OR = 32.0, 95% CI: 5.5–297.8) (*p* = 3 × 10^−5^) IFX TL are directly correlated to ALB levels (*p* = 0.0033) and inversely correlated with CRP (*p* = 0.0008) and fCal levels (*p* = 0.025). Patients with IFX TL <3.11 μg/mL had lower ALB (*p* = 0.03) and higher CRP (*p* = 3.6 × 10^−5^) and fCal (*p* = 0.0014) levels compared to patients who had higher IFX TL CRP (*p* = 0.0031) and fCal (*p* = 0.00017) levels at week 14 was also significantly associated with week 54 sustained remission. CRP levels were significantly associated with IFX TL <3 μg/mL in multivariate logistic regression (*p* = 0.0065)	
Chi et al. (2018) [45]	IFX	STD (4 dose categories)	Cross-sectional analysis of prospective observational study	223	^a^ 18.5 (4.4) yrs WT: NR	CD, UC, IBD-U	Combination therapy Age, sex, BMI, CRP, and IFX dose	Multi-linear regression model Patients currently on combination therapy had a higher IFX levels (17.00 ± 1.33 μg/mL) than those currently on IFX monotherapy (13.18 ± 1.26 μg/mL), IFX levels ≥3.5 μg/mL were associated with sustained response. 8.3% of patients on combination therapy had IFX levels <3.5 μg/mL compared to 27.3% of patients on monotherapy. (adjusted OR = 0.13, 95% CI: 0.04–0.39) (*p* = 0.01) There was a significantly lower risk of ATI in patients on combination therapy (9.5%) than in patients on monotherapy (20%) (OR = 0.3, 95% CI: 0.1–0.7) (*p* = 0.01) Patients with CRP levels >0.5 mg/dL had lower IFX levels (12.03 μg/mL) compared to patients with CRP ≤ 0.5 mg/dL (*p* = 0.03) Age was positively associated with IFX levels (*B* = 0.31, *p* = 0.04)	
Ungar et al. (2018) [61]	IFX	STD	Retrospective Cohort study	63	^a^ 14 (11.75–16) yrs WT: NR	CD, UC	PCDAI, PUCAI, HBI, CRP	Spearman correlation coefficient IFX TL negatively correlated with PCDAI (rho = −0.380, *p* < 0.0001) IFX TL negatively correlated with PUCAI (rho = −0.308, *p* = 0.0001) IFX TL negatively correlated with HBI (rho = −0.33, *p* < 0.0001) Patients in clinical remission had higher median IFX TLs (4 μg/mL) than patients with active disease (2.25 μg/mL) (*p* = 0.0001) Patients with normal CRP levels had higher median IFX TL (3.3 μg/mL) than patients with elevated CRP (2.7 μg/mL) (*p* = 0.02)	IFX TL have a negative correlation with PCDAI, PUCAI and HBI Normal CRP was positively associated with IFX TL than those with elevated CRP
Ohem et al. (2017) [60]	IFX	Median dose/kg: 6.5 (5.2–8.3) mg	Prospective observational study	65	^a^ 14 (11.4–16.4) yrs WT: NR	CD	ATI, CRP, ESR, CPT	Linear mixed model CRP (≤5 mg/L) and IFX level > 1.1 ug/mL (OR = 3.096, 95% CI: 1.394–6.874) (*p* = 0.005) ESR (≤200 mm/h) and IFX level > 2 ug/mL (OR = 2.872, 95% CI: 1.273–6.477) (*p* = 0.011) fCal (≤100 ug/g) and IFX level 3.5 ug/mL (OR = 3.333, 95% CI: 1.324–8.387) (*p* = 0.011) ATI (> 30 ng/mL) associated with IFX level (<30 ng/mL) (OR 0.027, 95% CI: 0.009–0.077)	CRP, ESR, ATI and fCal are negatively associated with IFX levels
Rolandsdotter et al. (2017) [25]	IFX	Mean dose/kg: 6.4 ± 1.7 mg Mean interval: 44.8 ± 11.2 days	Retrospective cohort study	45	^b^ 16 (7–18) yrs WT: NR	CD, UC	CRP, ESR, ALB, PUCAI and PCDAI	Linear Regression Mean IFX TL (7.2 μg/mL) higher in remission compared to TL in active disease (4.5 μg/mL) (*p* < 0.05) CRP levels negatively correlated with IFX TL (*p* = 0.0084, r^2^ = 0.0491) ESR negatively correlated with IFX TL (*p* = 0.0035, r^2^ = 0.1388) PUCAI and PCDAI negatively correlated with IFX TL (*p* = 0.0259, r^2^ = 0.0687) ALB positevely correlated with IFX levels (*p* = 0.0005, r^2^ = 0.2182).	CRP, ESR, PUCAI, PCAI were negatively correlated with IFX levels, while ALB was positively correlated with IFX level
Minar et al. (2016) [78]	IFX	STD + TDM	Retrospective cohort	75	^c^ 13 (4) yrs WT: NR	CD	IFX dose, IFX dosing frequency, ESR	Multiple Regression analysis ESR ≥ 15 mL/h was significantly associated with undetectable IFX TL (AUC = 0.70, 95% CI: 0.59–0.81) (*p* < 0.01) Undetectable IFX conc. associated with elevated serum biomarkers (ESR and albumin) (*p* < 0.01) Patients with undetectable IFX TL had significantly lower hematocrit comparted to patients with detectable IFX TL (*p* = 0.015) CRP, ALB, and platelet count preinduction were not associated with TDM outcomes in first year of therapy.	
Hämäläinen et al. (2013) [54]	IFX	STD	Prospective cohort study	37	^b^ 14 (5.6–18) yrs ^b^ 43.5 (19.6–67.1) kg	CD, UC	IFX dose and interval, CRP	Linear Regression Analysis Week 2 and week 6 IFX TL were significantly associated with total IFX dose, patients with lower body WT had lower IFX TL. (week 2: *p* < 0.001, week 6: *p* < 0.05) fCal >1000 mg/g in induction phase was correlated with lower IFX TL (4 ug/mL) than fCal <1000 mg/g (IFX TL 20 ug/mL) (*p* < 0.005) Shortening IFX dose intervals in maintenance phase was associated with higher IFX TL (*p* = 0.0002 for dose 5 mg/kg; *p* = 0.0013 for dose 10 mg/kg)	
Colman et al. (2024) [31]	IFX	Mono Cohort: STD Combo Cohort: STD + AZA	Mono Cohort: REFINE prospective cohort Combo Cohort: TISKids randomized controlled trial	128	^a^ 14 (11.1–16.0) yrs ^a^ 45.5 (33.2–55.8) kg	CD	ESR, ALB, WT	Parameter (%RSE) CL (L/day) 0.0151 V1 (L) 4.44 V2 (L) 1.94 Q (L) 0.000953 Covariate WT WT on CL 0.603 ALB on CL −0.731 ESR on CL 0.151 Model Equations Not provided	
Clemente et al. (2024) [30]	IFX	1. CD: 5 mg/kg at weeks 0, 2, 6 then every 8 weeks 2. Moderate UC: 10 mg/kg at weeks 0, 2, 6 then every 8 weeks 3. Severe UC: 10 mg/kg 0, 1, 3, and 7 then every 8 weeks	Observational cohort	30	^b^ 13 (1.3–16) yrs ^b^ 39.1 kg (9.6–74)	CD, UC	WT, ESR, fCal, SNP rs1048610 (ADAM17), and ALB	Parameter (RE%) *θ*Geno (L/h) AA = 6.6 × 10^−3^ AG = 5.5 × 10^−3^ GG = 8.1 × 10^−3^ *θ*Vc (L) 0.6750 *θ*Vp (L) 1.19 *Q* (L/h) 2.9 × 10^−3^ Covariate WT *θ*WT 0.36 *θ*fCal 13.7 × 10^−3^ *θ*ESR 62.8 × 10^−3^ *θ*ALB 7.39 Model Equations $CL=\theta Geno\times {\left(\frac{WT}{46.4}\right)}^{\theta WT}\times \left(1+\theta ESR\right)\times \frac{ESR}{27}\times \left(1+\theta fCal\right)\times \frac{fCal}{99.8}$ $Vc=\theta Vc\times \left(\frac{WT}{46.4}\right)$ $Vp=\theta Vp\times \left(\frac{WT}{46.4}\right)$ $Q=\theta Q\times {\left(\frac{ALB}{4.3}\right)}^{\theta ALB}$	
Maximova et al. (2023) [49]	IFX	Four doses of 10 mg/kg weekly	Prospective cohort	28	Early ^b^ 6.5 (0.6–17) yrs WT: NR Standard ^b^ 8 (1.1–17) yrs WT: NR	Acute Intestinal and Liver GVHD	WT	Parameter (%RSE) CL (L/day) 0.146 V(L) 3.43 Covariate WT WT on CL 0.75 WT on V 1 Model Equations $Cli=0.146*{\left(\frac{WTi}{26.2}\right)}^{0.75}$ $Vi=3.431*{\left(\frac{WTi}{26.2}\right)}^{1}$	Early: IFX use in first three days of steroids (first line) Standard: IFX in steroid refractory or second phase (second line)
Chung et al. (2023) [29]	IFX	^a^ Baseline dose: 6.58 mg/kg (5.92–7.36) ^a^ Baseline interval: 6 weeks (4–8)	Retrospective cohort	85	^a^ 12.7 (10.1–14.6) yrs ^a^ 37.4 (28–49.8) kg	CD	WT, ALB, CRP, Sex, ATI	Parameter (RE%) CL (L/day) 0.299 (1.3%) V1 (L) 3.3 (1.1%) V2 (L) 1.21 (9.6%) Q (L/day) 0.0697 (4.4%) Covariate WT WT on CL 0.75 (Fixed) ALB on CL −0.806 (7.9%) CRP on CL 0.0281 (20.9%) Sex on CL(F) −0.129 (26.7%) ATI on CL(+) 0.126 (39.3%) WT on V1 1 (Fixed) WT on V2 0.75 (Fixed) WT on Q 1 (Fixed) Model Equations Not provided.	
Whaley et al. (2023) [35]	IFX	^a^ Initial dose: 9.9 (9.3–10.3)	Prospective cohort	38	^a^ 14.5 (4–18) yrs ^a^ 52.6 (42.4–64.7) kg	UC	WT, ALB, ATI, WBC, CRP, PLT	Parameter (%RSE) CL (L/h) 0.0112 V1(L) 2.69 Q(L/h) 0.00731 V2(L) 2.8 Covariate WT ALB on CL −0.718 ATI on CL 4.61 WBC on CL 0.235 WT on V1 0.666 PLT on V2 −2.11 WT on V2 0.666 CRP on Q −0.592 Model Equations: $CL=0.0112\times {\left(\frac{ALB}{3.5}\right)}^{-0.718}\times \left(1+\left(ATI\times 4.61\right)\right)\times {\left(\frac{WBC}{7.9}\right)}^{0.235}$ $V1=2.69\times {\left(\frac{WT}{65}\right)}^{0.666}$ $Q=0.00731\times {\left(\frac{CRP}{0.6}\right)}^{-0.592}$ $V2=2.8\times {\left(\frac{WT}{65}\right)}^{0.666}\times {\left(\frac{PLT}{328.49}\right)}^{-2.11}$	
Funk et al. (2021) [18]	IFX	^a^ Dose -IBD 7.7 (6.2–9.4) -JIA 9.7 (8.5–10.8) -Uveitis 10.5 (7.5–12) ^a^ Interval -IBD 6 (4–8) weeks -JIA 4 (4–4.8) weeks -Uveitis 4 (4–5.5) weeks	Prospective cohort	97	^a^ 16 (5–21) yrs WT: NR	CD, UC, JIA, Uveitis	ALB, ESR, ATI	Parameter (%RSE) Cl (L/kg/d) 0.00231 (47.2) V1 (L/kg) 0.0542 (FIX) Q (L/kg/d) 0.00352 (FIX) V2 (L/kg) 0.0292 (FIX) Covariate WT ALB on CL −1.8 (25.3) ESR on CL 0.687 (39.6) ATI on CL 1.04 (18.2) Model Equations: $CL=0.00231\times {\left(\frac{ALB}{4.3}\right)}^{-1.8}\times {\left(\frac{ESR}{9}\right)}^{0.069}\times 1.04^{ATI}\times \exp\left(IIVcl\right)$ ATI = 1 in patients with ATI ATI = 0 in patients without ATI	
Xiong et al. (2021) [36]	IFX	^a^ Dose 6.1 mg/kg (5.2–7.1)	Prospective cohort	78	^c^ 13 (3.7) yrs ^a^ 41 (28–56.8)	CD	WT, ALB, ATI, ESR, nCD64	Parameter (%RSE) Cl (L/h/65 kg) 0.0138 (5.7) V1 (L/65 kg) 2.97 (4.5) Q (L/h/65 kg) 0.0095 (FIX) V2 (L/65 kg) 2.84 (3.6) Covariate WT: WT on CL 0.5941 (10.2) ALB on CL −1.07 (9.6) ATI on CL 0.134 (9.6) ESR on CL 0.101 (27.7) nCD64 on CL 0.168 (16) WT on V1 0.55 (20.2) WT on V2 0.586 (FIX) WT on Q 1 (FIX) Model Equations: ${CL=CL}_{pop}\times {\left(WT/65\right)}^{0.594}\times {\left(ALB/3.5\right)}^{-1.07}\times {\left(ATI/22\right)}^{0.134}\times {\left(ESR/9\right)}^{0.101}\times {\left(\frac{nCD64}{4.6}\right)}^{0.168}$ $V1={V1}_{pop}\times {\left(WT/65\right)}^{0.55}.$	
Bauman et al. (2020) [28]	IFX	STD	Retrospective cohort	228	^c^ 14.5 ± 3.6 yrs ^c^ 56.25 ± 22 kg	CD, UC	WT, ALB, ATI, ESR	Parameter (%RSE) CL (L/h/65 kg) 0.0122 (3.4) V1 (L/65 kg) 3.52 (FIX) Q (L/h/65 Kg) 0.0095 (FIX) V2 (L/65 kg) 1.9 (FIX) Covariate WT WT on CL 0.698 (8.5) ALB on CL −1.1 (10.9) ATI on CL 1.18 (2) ESR on CL 0.109 (28.6) WT on V1 0.829 (FIX) WT on V2 0.586 (FIX) WT on Q 1 (FIX) Model Equations: ${CL}_{ind}={CL}_{pop}\times {(\mathrm{WT}/65)}^{0.7}\times {(\mathrm{ALB}/3.5)}^{-1.1}\times {(\mathrm{ESR}/9)}^{0.11}$$\times 1.18^{\mathrm{ATI}}$	
Kimura et al. (2020) [79]	IFX	STD	Prospective cohort	15	^d^ 44 (13–76) yrs WT: NR	CD, UC	-	Two models: PK Parameter: Crohn’s Disease: Cl (L/d) 0.35 (0.21–0.44) V1 (L) 2.97 (1.96–5) Q (L/d) 1.2 (1.1–1.5) V2 (L) 0.137 (0.09–0.27) UC: Cl (L/d) 0.45 (0.22–0.8) V1 (L) 2.25 (1.6–3.2) Q (L/d) 7.1 (6.96–7.32) V2 (L) 3.7 (3.29–4.06) Model Equations: Used Fasanmade et al. model equations	
Petitcollin et al. (2018) [34]	IFX	STD	Observational cohort	20	^b^ 13.4 (6.7–16.2) yrs ^b^ 36.9 (25.8–50.6) kg	CD	ALB, Clearance variation magnitude	PK Parameters (RSE%): Fixed effects CL_base_ (L/d^−1^) 0.289 (9) V (L) 4.86 (11) ALB on CL −2.33 (37) Base-risk 4.07 (15) Beta 0.0484 (16) CL_var_ 1.01 (28) Model Equations: ${risk}_{i}\left(t\right)=-{baserisk}_{i}+{beta}_{i}\left(t\right)$ $logit\left[{risk}_{i}\left(t\right)\right]=\frac{{exp}^{{risk}_{i}(t)}}{1+{exp}^{{risk}_{i}(t)}}$ ${CL}_{i}\left(t\right)={CL}_{{base}_{i}}\times {{CL}_{{var}_{i}}}^{logit[{risk}_{i}(t)}$	*Abbreviations*
Kevans et al. (2018) [33]	IFX	STD	Retrospective cohort	36	^b^ 28 (11.6–64.9) yrs ^b^ 61 (44–104) kg	UC	WT, ALB, ATI, Time Dependent CL	PK Parameters (SE%): Fixed effects CL (L/d) 0.368 (0.5) V1 (L) 3.3 (0.2) Q (L/d) 0.308 (0.1) V2 (L) 3.42 (0.5) Covariate WT: WT on CL 0.709 (0) ALB on CL −0.445 (2.6) ATI on CL −0.0373 (4.9) WT on V1 0.64 (0) WT on V2 0.991 (0) WT on Q 1.52 (0) Cl_time_ 0.105 (0.4) KINC 0.138 (25.5) Model Equations: No equations provided	
Van de Casteele et al. (2018) [80]	IFX	Study 1: 5 mg/kg Study 2 IFX: 5 mg/kg or Placebo Saline 5 mL/kg	Two randomized, prospective, clinical trials	16 + 54	^a^ 2.9 (1.3–4.4) yrs ^a^ 14 (10–18.2) kg	Kawasaki Disease	Prior IVIG	Parameter CL (L/d) 0.117 V1 (L) 0.758 V2 (L) 0.973 Q (L/d) 0.683 Covariate Model: V2 × Prior IVIG −0.466 Model Equations: $CL=0.117\frac{L}{d}\times {\left(\frac{WT}{14\;\mathrm{kg}}\right)}^{0.75}$ $V1=0.101L\times {\left(\frac{WT}{14\;\mathrm{kg}}\right)}^{1}$ $V2=0.962L{\left(\frac{WT}{14\;\mathrm{kg}}\right)}^{1}\times \left(1+IVGF\times -0.497\right)$ $Q=0.692\frac{L}{d}*{\left(\frac{WT}{14\;\mathrm{kg}}\right)}^{0.75}$ IVIG = 1 for patients who received IVIG INF before IFX	
Fasanmade et al. (2011) [32]	IFX	STD	Randomized, multi-centre, open label study	112	^b^ 13 (6–17) yrs ^b^ 42 (20.4–97.7) kg	CD	WT, ALB	Parameter (RSE%): Cl (mL/kg/d) 5.43 (2.8) V1 (mL/kg) 54.2 (2.1) Q (mL/kg/d) 3.52 (20.1) V2 (mL/kg) 29.2 (7.0) Covariate WT ALB on CL −1.22 (20.4) WT on CL −0.34 (25.6) WT on V1 −0.171 (40.2) WT on V2 −0.414 (32.6) Model Equations: $\mathrm{CL}=5.42\;\times \;{(\frac{\mathrm{WT}}{65}\;)}^{-0.313}\;\times \;{\left(\frac{\mathrm{ALB}}{4.1}\right)}^{-0.855}$$\times \;{\left(1.29\right)}^{\mathrm{ATI}}\;\times \;{\left(0.863\right)}^{\mathrm{IMM}}$ $Vc=52.4\times ({\frac{WT}{65})}^{-0.233}$ $Vp=19.6\times {\left(\frac{WT}{65}\right)}^{-0.588}$ Q=2.26	

ADA: Adalimumab; ATI: antibodies to infliximab; AUC: area under the curve; AZA: azathioprine; Base-risk: intercept-risk of ATI, Beta: slope-risk of ATI, BMI: body mass index; CL: Clearance; CL_base_: CL at baseline, CL_var_: CL variation magnitude; Cl_time_: time-dependent CL, CD: Crohn’s disease; CRP: C-reactive protein; ESR: erythrocyte sedimentation rate; fCal: fecal calprotectin; GVHD: graft versus host disease; HBI: Harvey-Bradshaw Index IFX: infliximab; INF: infusion; IBD-U: inflammatory bowel disease—unspecified; IIV: Interindividual variability; IVIG: Intravenous immunoglobulin; JIA: Juvenile idiopathic arthritis; KINC: rate constant of clearance increase with ATI; NR: not reported; PCDAI: pediatric Crohn’s disease activity index; PLT: platelets; PRO: Proactive dosing using individual PK profile; PUCAI: pediatric ulcerative colitis activity index, Q: intercompartmental clearance; SNP: single nucleotide polymorphism; STD: standard dosing and interval and/or dosing at discretion of treating physician; TDM: therapeutic drug monitoring; TL: trough levels; UC: ulcerative colitis; Vd: volume of distribution; WBC: white blood cells; WT = weight. ^a^ Median (IQR); ^b^ Median (range); ^c^ Mean (SD); ^d^ Mean (Range).

## 5. Conclusions

Our study provides valuable insights into the PK of IFX in children with IBD. We identified significant associations between IFX and albumin concentration, IFX dose, and treatment phase. Albumin concentrations should be carefully assessed when optimizing IFX therapy in paediatric patients with IBD. The high proportion of subtherapeutic IFX concentrations, especially during induction, emphasizes the need for personalized monitoring strategies. We recommend more frequent TDM during this phase in order to achieve the target attainment and reduce the risk of treatment failure. Moreover, it is recommended to consider higher initial doses or more frequent dosing for patients with low albumin levels due to its significant (positive) association with IFX concentrations. Our findings support the development of personalized treatment strategies and a more individualized approach is needed based on individual characteristics, disease severity and treatment response.

## Figures and Tables

**Table 1:** Characteristics of 115 children with IBD and IFX therapy. Baseline characteristics are at the time of the first IFX trough concentration. Data are presented as mean |SD|, median [IQR] or proportions n with corresponding percentages.

Baseline Characteristics	Number
Total nr. of IFX concentrations	417
Total nr. of paediatrics	115 (100%)
Sex - Female - Male	Sex 51 (44.3%) 64 (55.7%)
Age (years)	14.5 [5–17]
Height (cm)	161.9 |16.5|
Weight (kg)	51.4 |17.1|
BMI (kg/m^2^)	19.0 [IQR: 16.8–21.6]
Diagnosis - Crohn’s disease - Ulcerative colitis - Inflammatory bowel disease unclassified - Acute severe ulcerative colitis - Very early onset inflammatory bowel disease	81 (70.4%) 27 (23.5%) 3 (2.6%) 2 (1.7%) 2 (1.7%)
Nr. patients receiving co-medication - Azathioprine (AZA) - Methotrexate (MTX) - Mercaptopurine (6-MP) - AZA + Mesalazine - None	78 (67.8%) 7 (6.1%) 2 (1.7%) 21 (18.3%) 7 (6.1%)
Use of corticosteroids - Prednisolone - None	8 (7.0%) 107 (93%)
Median serum concentrations - IFX (μg/mL) - Albumin (g/L) - CRP (mg/L) - Antibodies to IFX (number/percent) - fCal (mg/kg)	8.7 [0.05–74] 45 [24–55] 0.8 [0.15–148] 17.4% 239 [3–13,590]
Smoking - Yes - No	6 (5.2%) 109 (94.8%)
Dose of IFX (mg/kg) - 5 - 8 - 10 - 15	56 (33.3%) 1 (0.5%) 77 (63.8%) 3 (2.4%)

Characteristics depicted as number (proportion of total number in %); [range]; |SD|.

**Table 2:** Linear mixed model analysis of associations between clinical, physical and biochemical parameters and serum IFX concentration in paediatric patients with IBD. Abbreviations: SE: standard error; CI: confidence interval.

Variable	b (SE)	95% CI	*p*-Value	F-Statistics	*p*-Value
Sex - Male - Female	reference 1.737 (1.946)	- −2.123–5.597	- 0.374	0.797	0.374
Age	0.099 (0.299)	−0.493–0.691	0.742	0.109	0.742
Height	−0.006 (0.069)	−0.143–0.131	0.928	0.008	0.928
Weight	−0.037 (0.067)	−0.170–0.096	0.579	0.310	0.579
BMI	−0.166 (0.299)	−0.757–0.425	0.579	0.309	0.579
Diagnosis - CD - UC	reference −0.088 (2.233)	- −4.523–4.348	- 0.969	0.002	0.969
IFX dose - 5 mg/kg - 10 mg/kg - 15 mg/kg	reference 6.534 (1.440) 9.424 (5.201)	- 3.701–9.367 −0.854–19.701	- <0.001 0.072	9.904	<0.001 *
IFX phase Induction maintenance	reference −4.922 (1.229)	- −7.338–−2.507	- <0.001	16.046	<0.001 *
Albumin	0.388 (0.188)	0.019–0.757	0.039 *	4.270	0.039 *
CRP	−0.006 (0.048)	−0.101–0.089	0.902	0.015	0.902
fCal	0.001 (4.8 × 10^−4^)	−2.8 × 10^−4^–0.002	0.171	1.886	0.171
Concurrent medication ^^^ - Thiopurines (AZA/6-MP) - MTX - Mesalazine	−2.536 (2.286) 2.333 (3.933) −4.132 (2.526)	−2.003–7.706 −5.473–10.139 −9.151–0.888	0.270 0.555 0.106	1.231 0.352 2.674	0.270 0.555 0.106

Estimates of fixed effects (b with SE, including 95% CI) are shown with corresponding *p*-values derived from linear mixed model analyses. The *p*-values in bold are significant results. Variables were univariately analysed as fixed effects (either as a factor or as a covariate) with a random effect applied to individual subjects. The dependent variable was serum IFX concentration. Linear mixed models were used to assess the correlation between potential covariates and IFX serum concentrations. The statistical significance was set at *p*-value ≤ 0.05 *. ^^^ reference to patients not using each medication type.

## Data Availability

Data is available upon reasonable request via de corresponding author.

## Appendix A

Search query for PubMed for infliximab pharmacokinetics children

“((“Infant, newborn”[Mesh] OR Infant [Mesh] OR Child [Mesh] OR Adolescent [Mesh] OR neonat*[tiab] OR baby[tiab] OR babies[tiab] OR newborn*[tiab] OR postneonat*[tiab] OR postnat*[tiab] OR perinat*[tiab] OR nicu[tiab] OR new-born*[tiab] OR neo-nat*[tiab] OR neonat*[tiab] OR prematur*[tiab] OR pre-matur*[tiab] OR postmatur*[tiab] OR post-matur*[tiab] OR preterm*[tiab] OR pre-term*[tiab] OR child*[tiab] OR infan*[tiab] OR adolescen*[tiab] OR pediatri*[tiab] OR paediatr*[tiab] OR boy[tiab] OR boys*[tiab] OR girl*[tiab] OR youth*[tiab] OR toddler*[tiab] OR teen*[tiab] OR pube*[tiab] OR *school*[tiab] OR suckling*[tiab] OR picu[tiab] OR youngster*[tiab] OR kindergart*[tiab] OR kid[tiab] OR kids[tiab] OR playgroup*[tiab] OR play-group*[tiab] OR prepube*[tiab] OR preadolescen*[tiab] OR junior high*[tiab] OR senior high*[tiab] OR young people*[tiab] OR minors [tiab]) AND (“Pharmacokinetics”[Mesh] OR pharmacokinetic*[tiab] OR “drug kinetic*”[tiab] OR ADME*[tiab] OR LADMER[tiab] OR (absorption[tiab] AND distribution[tiab] AND metabolism[tiab] AND elimination[tiab]) OR “pharmacokinetics” [Subheading]) AND (“Infliximab “[Mesh] OR Infliximab [tiab] OR Inflectra [tiab] OR Remicade [tiab] OR Renflexis [tiab] OR infliximab-abda [tiab] OR “infliximab abda”[tiab]))”
